# Supplementary material for: LBNP: Learning features between neighboring points for point cloud classification
Source: PLoS One. 2025 Jan 6;20(1):e0314086. doi: 10.1371/journal.pone.0314086 (PMC11703072; doi:10.1371/journal.pone.0314086)
Supplement: Attachment — Submitted filename: Response to Reviewers#2.docx [file pone.0314086.s001.docx]

**Response to Comments on the Manuscript:**

**“LBNP: Learning features between neighboring points for point cloud classification”**

-------------------------------------------------------------------------------------------------------

The authors gratefully acknowledge the editors and the anonymous reviewers for their constructive comments. We have made a comprehensive revision for our previous manuscript. Specially, any revisions are highlighted using the "Track Changes" function in Microsoft Word. Please refer to the point by point response. Thank you for your time.

**Response to comments by Reviewer #2**：

We would like to gratefully thank the reviewer for his/her constructive comments and recommendations for improving the paper. A point-by-point response to the interesting comments raised by the reviewer follows.

**Major comment:**

**Point 1. The percentage symbols in Equations 4, 5, 7, and 8 are misaligned. It is recommended to adjust the size of the percentage symbols to make them look more coordinated.**

**Response 1：**We revised the formula expression to make it clearer and more aesthetically pleasing. Specifically refer to the Revised Manuscript with Track change or following sentence:

$$\tilde{A}=(\tilde{a})_{i,j}=Q\cdot K^{T}$$

$$\begin{matrix} (\bar{a})_{i,j}=softmax(\tilde{a}_{i,j})=\frac{exp(\tilde{a}_{i,j})}{\sum_{k} exp(\tilde{a}_{k,j})+C} \\ A_{g}=a_{i,j}=\frac{\bar{a}_{i,j}}{\sum_{k} \bar{a}_{i,k}} \end{matrix}$$

$$A_{l}=Norm(\tilde{V}_{k}M_{k}^{T})$$

$$\bar{V}_{k}=A_{l}M_{v}$$

**Point 2. It is suggested that the author introduces more about the connection between different methods and their own work in the related work section. Explain how your method finds innovation among many methods, rather than merely listing the contents of others' work extensively.**

**Response 2：**Thank you for your suggestion. We have added content to enhance the relevance of the related work to this Manuscript, to highlight its relevance. Specifically refer to lines 122-127,165-168,174-176 of the Revised Manuscript with Track change or following red sentence:

122-127：

Generally, local aggregation operators can be divided into two categories: convolution- and graph-based methods. Among the convolution-based methods, PointCNN[14] uses x-transformation to learn the weights and order of neighborhood points to ensure that the point cloud can perform convolution operations. KPConv[15]associates the weight matrix with a predefined kernel point in 3D space to adapt it to the uneven distribution of local points. PAConv[16] constructs a position-adaptive convolution operator with a dynamic kernel, which combines weight matrices in a weight library, and uses MLP to learn weight coefficients from relative point positions.Among graph-based methods, DGCNN[17], as a pioneering method, aggregates neighboring points in the feature space on a dynamically updated graph at each layer. CurveNet[18]grouped line segments connected into lines in a point cloud to expand the receptive field and explore useful geometric information. Most of these methods follow the basic paradigm of PointNet++, that is, the relationship between adjacent points and center points is constructed by absolute or relative coordinates, and then other simple or complex operations are used to obtain more explicit local geometric features. We believe that local features are not only implied in the center point and adjacent points, but also there is fine geometric information between adjacent points. Therefore, our proposed PLAB module differs from the above or other approaches. We attempt to enhance the information representation of neighborhood aggregation by exploring the relationship between neighborhood points used to aggregate features and exert a strengthening effect on different types of local modules.

165-168,174-176：

In the above studies, global attention mechanisms cannot provide more refined local geometric capture, while local attention mechanisms cannot guarantee global context sensitivity and must use other auxiliary methods to obtain global perception. We were inspired by 3DPCT[26],it introduced a dual attention mechanism that downsamples the original point cloud and uses different neighborhood sizes to obtain local multi-scale information; it then performs global self-attention on the aggregated multi-scale information and uses the calculated self-attention map to further weight the multi-scale information to improve the local feature extraction ability. Finally, it is fused with the global module, which uses an affine matrix to enhance channel information expression as the final feature output. In contrast, The attention mechanism we propose is fully implemented within the Transformer architecture to achieve both global and local attention simultaneously. Essentially, the relevance score in the dot product attention score map is used to index the local points in the feature space, and the linear attention mechanism is used to enhance the expression of local points in the feature space, which simplifies the model and improves the global and local representation of point cloud features.

**Point 3. It is recommended to provide more detailed explanations for the formulas in the text.**

**Response 3：**We have provided a more detailed explanation of the formulas in the Manuscript. Specifically refer to lines 289-330 of the Revised Manuscript with Track change or following red sentence:

Where $\mathop{A}\limits^{\sim}$is the attention score matrix obtained by computing the dot product of $Q$ and $K$.

We normalize the weights and assign them to the score matrix $\tilde{A}$ as follows:

$$\begin{matrix} (\bar{a})_{i,j}=softmax(\tilde{a}_{i,j})=\frac{exp(\tilde{a}_{i,j})}{\sum_{k} exp(\tilde{a}_{k,j})+C} \\ A_{g}=a_{i,j}=\frac{\bar{a}_{i,j}}{\sum_{k} \bar{a}_{i,k}} \end{matrix}$$

Where the *softmax* normalization is used here to normalize the attention score matrix $\tilde{A}$, and then *l_1_-norm* is applied for further normalization.

Next, we select the Top-K attention scores from matrix *A_g_* to obtain *V_k_*, which corresponds to the selected $V$values. *V_k_* is then used as input for the local module:

$$\tilde{V}_{k}=V-V_{k}$$

where *V_k_* is the nearest neighbor feature in feature space obtained by score matrix indexing, local features $\tilde{V}_{k}$ are obtained by establishing the relative relationship.

$$A_{l}=Norm(\tilde{V}_{k}M_{k}^{T})$$

$$\bar{V}_{k}=A_{l}M_{v}$$

Where *M_k_* and *M_v_* are learnable linear layers, and after calculating *A_l_*, steps (5) are still executed. $\bar{V}_{k}$ is the computed result of local External-Attention.

We obtain the local attention feature matrix, aggregate the features, and compute the global attention as follows:

$$\bar{V}=max(\bar{V}_{k})$$

Where the local feature $\bar{V}_{k}$ is aggregated into feature $\bar{V}$ after maximum pooling.

$$F_{sa}=A_{g}\cdot\bar{V}$$

the local aggregated features are weighted by the attention score matrix *A_g_* to obtain the global self-attention *F_sa_*.

The final output is obtained through the bias module:

$$F_{out}=BR(LBR(F_{s}-F_{sa})+F_{s})$$

Where $LBR$stands for *Linear*, *BatchNorm*, and *ReLU*. This part is used to enhance the model's feature learning.

**Point 4. The images inserted in the text are mostly of insufficient clarity; it is recommended to replace them with higher-resolution images.**

**Response 4：**Thank you for your suggestion. We have modified some unclear images and tried to make them more visually appealing, as shown in Fig 4. We have also removed Fig 1 due to copyright issues. Specifically refer to line 383 of the Revised Manuscript with Track change or following image:
