# Supplementary material for: LBNP: Learning features between neighboring points for point cloud classification
Source: PLoS One. 2025 Jan 6;20(1):e0314086. doi: 10.1371/journal.pone.0314086 (PMC11703072; doi:10.1371/journal.pone.0314086)
Supplement: Attachment — Submitted filename: Reviewer Attachments for Manuscript.docx [file pone.0314086.s002.docx]

1.There are several spelling and grammar errors in the text, such as the phrase "performed we this module" in the abstract. These details need to be meticulously proofread to prevent unnecessary mistakes that could impact the quality of the paper.

2.It has been noted that the images in the paper, particularly Figure 4, lack clarity. It is recommended to ensure that all images are of high resolution to facilitate easier viewing for readers.

3.In Table 1, the title and body are misaligned in several areas, which affects readability. Furthermore, the term "point" is redundant and should be eliminated from the table.

4.The explanations for formulas 4, 5, 7, and 8 lack sufficient detail. It is recommended to provide more comprehensive explanations of the derivation and underlying principles of these formulas. This will help ensure that readers can clearly grasp the derivation process and the physical significance of each step.

5.Although the "Related Work" section references relevant studies, the analysis primarily consists of a basic enumeration of their contents. It is advisable to emphasize the distinctions and innovations of your method in comparison to existing approaches to further strengthen the paper's persuasiveness.

6.Although the experimental results indicate that the method performs well on certain datasets, a more in-depth discussion is necessary regarding specific cases (e.g., the reasons why the method underperforms compared to DCNet on the FG3D dataset). Clarifying these differences will enhance readers' understanding of the method's strengths and weaknesses.

7.Some paragraphs transition rather abruptly, particularly between the introduction of the methods and the experiments section. Incorporating transitional sentences can enhance the overall flow and coherence of the paper's structure.
