# Supplementary material for: LBNP: Learning features between neighboring points for point cloud classification
Source: PLoS One. 2025 Jan 6;20(1):e0314086. doi: 10.1371/journal.pone.0314086 (PMC11703072; doi:10.1371/journal.pone.0314086)
Supplement: Attachment — Submitted filename: Response to Reviewer.docx [file pone.0314086.s003.docx]

**Response to Comments on the Manuscript:**

**“LBNP: Learning features between neighboring points for point cloud classification”**

-------------------------------------------------------------------------------------------------------

The authors gratefully acknowledge the editors and the anonymous reviewers for their constructive comments. We have made a comprehensive revision for our previous manuscript. Specially, any revisions are highlighted using the "Track Changes" function in Microsoft Word. Please refer to the point by point response. Thank you for your time.

**Response to comments by Reviewer**：

We would like to gratefully thank the reviewer for his/her constructive comments and recommendations for improving the paper. A point-by-point response to the interesting comments raised by the reviewer follows.

**Point 1: There are several spelling and grammar errors in the text, such as the phrase "performed we this module" in the abstract. These details need to be meticulously proofread to prevent unnecessary mistakes that could impact the quality of the paper.**

**Response 1：**Thanks to your suggestion, we have carefully checked the whole text and corrected several grammatical and spelling errors. You can in Revised Manuscript with Track change.

**Point 2: It has been noted that the images in the paper, particularly Figure 4, lack clarity. It is recommended to ensure that all images are of high resolution to facilitate easier viewing for readers.**

**Response 2：**Thanks to your suggestions, we have replaced all the images with higher resolution images and Figure 4 has been redrawn with new tools to make it look better. You can view it below or in Revised Manuscript with Track change.

Fig. 1

Fig. 2

Fig.3

Fig. 4

**Point 3: In Table 1, the title and body are misaligned in several areas, which affects readability. Furthermore, the term "point" is redundant and should be eliminated from the table.**

**Response 3：**Thanks to your careful scrutiny, we've reworked the problems with the table 1. You can check it out below, as well as in the Revised Manuscript.

Table 1

| Methods | Input | OA (%) | Parameters | FLOPs |
| --- | --- | --- | --- | --- |
| Other Learning-based Methods | | | | |
| SO-Net[30] | 2048×3 | 90.9 | - | - |
| Point2Sequence[31] | 2048×3 | 92.6 | - | - |
| PointCNN[14] | 1024×3 | 91.7 | - | - |
| PointNet^†^[1] | 1024×3 | 89.2 | 3.47 M | 0.45 G |
| PointNet++(SSG) ^†^[2] | 1024×3 | 92.4 | 1.48 M | 0.87 G |
| PointNet++(MSG) ^†^[2] | 1024×3 | 92.7 | 1.75 M | 4.07 G |
| DGCNN^†^[17] | 1024×3 | 92.6 | 1.82 M | 2.43 G |
| DGCNN+Pnp-3D^†^[32] | 1024×3 | 92.5 | 1.93 M | 3.57 G |
| PointMLP^†^[33] | 1024×3 | 92.8 | 13.23 M | 15.73 G |
| DualMLP^†^[34] | 1024×3 | 93.1 | 14.32M | - |
| G-PointNet++^†^[35] | 1024×3 | 92.7 | - | - |
| Transformer-based Methods | | | | |
| GBNet^†^[36] | 1024×3 | 92.7 | 8.79 M | 9.86 G |
| Point Transformer^†^[37] | 1024×3 | 91.1 | 9.85 M | 18.40 G |
| PCT^†^[5] | 1024×3 | 92.4 | 2.88 M | 2.32 G |
| 3DGTN ^†^[38] | 1024×3,N | **93.3** | 5.12 M | 3.09 G |
| DCNet^†^[39] | 1024×3 | 92.4 | 2.21 M | 7.80 G |
| PointConT^†^[40] | 1024×3 | 92.9 | - | - |
| Ours | 1024×3 | **93.3** | 2.43 M | 5.60 G |

**Point 4: The explanations for formulas 4, 5, 7, and 8 lack sufficient detail. It is recommended to provide more comprehensive explanations of the derivation and underlying principles of these formulas. This will help ensure that readers can clearly grasp the derivation process and the physical significance of each step.**

**Response 4：**Thanks for your suggestion, I have provided more explanations for formulas 4, 5, 7, and 8. You can check it out below, as well as in the Revised Manuscript.

We compute the attention weights by matrix dot product using *query* and *key*:

$$\tilde{A}=(\tilde{a})_{i,j}=Q\cdot K^{T}$$

Where $\tilde{A}$ is the attention score matrix obtained by computing the dot product of *Q* and *K*. Specifically, based on the query vector *Q*, a weight distribution $\tilde{A}$ is obtained by calculating the similarity of all query vectors *Q* with all key vectors *K.*

We normalize the weights and assign them to the score matrix $\tilde{A}$ as follows:

$$\begin{matrix} (\bar{a})_{i,j}=softmax(\tilde{a}_{i,j})=\frac{exp(\tilde{a}_{i,j})}{\sum_{k} exp(\tilde{a}_{k,j})+C} \\ A_{g}=a_{i,j}=\frac{\bar{a}_{i,j}}{\sum_{k} \bar{a}_{i,k}} \end{matrix}$$

where *softmax* enhances the difference between the values through exponential operations, so that larger attention scores are given higher weights in the probability distribution, and the influence of smaller attention scores is suppressed, and then *l_1_-norm* is applied for further normalization to reduce the noise effect.

Next, we select the Top-K attention scores from matrix *A_g_* to obtain *V_k_*, which corresponds to the selected *V* values. *V_k_* is then used as input for the local module:

$$\tilde{V}_{k}=V-V_{k}$$

where *V_k_* is the nearest neighbor feature in feature space obtained by score matrix indexing, local features $\tilde{V}_{k}$ are obtained by establishing the relative relationship.

$$A_{l}=Norm(\tilde{V}_{k}M_{k}^{T})$$

$$\bar{V}_{k}=A_{l}M_{v}$$

where *M_k_* and *M_v_* are learnable linear layers. Since $\tilde{V}_{k}$corresponds to a neighborhood in the feature space and each feature vector affects *M_k_* and *M_v_*, a memory cell is formed that takes into account all local features. After computing *A_l_*, step (5) is still performed. Finally, $\bar{V}_{k}$ is the computed result of the local External-Attention.

**Point 5: Although the "Related Work" section references relevant studies, the analysis primarily consists of a basic enumeration of their contents. It is advisable to emphasize the distinctions and innovations of your method in comparison to existing approaches to further strengthen the paper's persuasiveness.**

**Response 5：**Thanks to your suggestion, we've added more differentiating descriptions to our related work to highlight the distinctiveness and innovation of our approach. You can check it out below, as well as in the Revised Manuscript.

**Point cloud neighborhood feature aggregation：**

In previous approaches, a widely used encoding approach is by constructing the coordinates of neighboring points relative to the center point, which brings benefits to the model in terms of position invariance and preservation of local structure[14-17]. However, we believe that such an encoding approach ignores the associations between neighboring points, because the local geometry exists not only in the relative relationship between neighboring points and the centroid, but as a whole in all pairs of points. In order to enhance the associations between point pairs, we attempt to enhance the representation of geometric information in the neighborhood by exploring the relationships between neighborhood points used to aggregate features, hence the proposed PLAB module.

**Point Cloud Transformer：**

In the above studies, the global attention mechanism fails to provide finer local geometric capture[5, 19, 20], while the local attention mechanism loses its long-range dependency[4], and the hybrid approach incorporates other complex modules or structures[21, 23-26]. However, it is easy to be overlooked that the global attention matrix is the neighborhood representation of points in the feature space, and we propose that DAL using the neighborhood features indexed by the attention graph can preserve the long-range dependence property inside the Transformer while accomplishing the local aggregation.

**Point 6: Although the experimental results indicate that the method performs well on certain datasets, a more in-depth discussion is necessary regarding specific cases (e.g., the reasons why the method underperforms compared to DCNet on the FG3D dataset). Clarifying these differences will enhance readers' understanding of the method's strengths and weaknesses.**

**Response 6：**Thank you for your insightful suggestions. Indeed, networks targeted for fine-grained point cloud classification tasks differ in their design goals from those for traditional point cloud classification tasks. For the traditional point cloud dataset (ModelNet40), the network usually needs to learn the global structure of the point cloud samples in order to perform classification efficiently. However, since fine-grained point cloud datasets (FG3D) have relatively small interclass sample differences and relatively large intraclass sample differences, this property motivates the network to have to pay more attention to the local detailed features in order to achieve more accurate classification. As shown in Fig.5, fine-grained point cloud classification differs significantly from traditional point cloud classification in terms of the focus of feature learning. In order to focus on the ModelNet40 dataset, our proposed network concentrates on learning the global structure of the point cloud samples for the classification task, and thus has a lower accuracy than DCNet on the FG3D dataset. However, it is worth noting that our net exhibits superior performance on the fine-grained dataset FG3D compared to existing networks such as PCT. This is mainly due to the innovative design of the dual attention layer in terms of local feature capture, which enables the network to extract some fine features more efficiently, thus jointly improving the coarse- and fine-grained classification accuracy. We also analyse this problem in the manuscript，you can check it out below, as well as in the Revised Manuscript.

Fig.5 Difference between coarse- and fine-grained levels.

Table 3 shows the classification accuracies of airplane, car and chair using our method. DCNet performs better on fine-grained datasets compared to our method, and its model focuses exclusively on local details because FG3D has greater intraclass sample variance, which requires that the model must be more attentive to the sample details, whereas ModelNet40 needs to be more attentive to the global features in order to achieve the interclass sample recognition, which also makes it perform poorly on coarse-grained datasets. Our method balances the two requirements well and achieves good results on both coarse- and fine-grained datasets, although it does not perform as well as DCNet on fine-grained datasets.

**7.Some paragraphs transition rather abruptly, particularly between the introduction of the methods and the experiments section. Incorporating transitional sentences can enhance the overall flow and coherence of the paper's structure.**

**Response 7：**The excesses of these two parts were indeed abrupt, we readjusted the transitional sentences to make the manuscript smoother. You can check it out below, as well as in the Revised Manuscript.

After describing the methodology, we test the accuracy of the proposed method on the ModelNet40 dataset and the FG3D dataset for both coarse- and fine-grained classification tasks, and compare it with a range of classical and state-of-the-art models to illustrate the advancement of our method. In addition, we insert the PLAB module into other models to illustrate its effectiveness. We also provide implementation details of the models, including hardware configuration and hyperparameter settings.
